# Note bloat

**DOI:** 10.1038/s41415-025-8604-8

**Published:** 2025-09-12

**Authors:** Shamir B. Mehta, Raj Rattan, Len D´Cruz

**Affiliations:** 41415436662001 Lead Clinical Adviser, Fitness to Practise, Regulation Directorate, General Dental Council, UK; Programme Director MSc in Aesthetic Dentistry, King´s College London, Faculty of Dentistry, Oral and Craniofacial Sciences, Guy´s Campus, London, UK; 41415436662002 Dental Director, Dental Protection Ltd, UK; 41415436662003 Head of BDA Indemnity, General Dental Practitioner, UK

## Abstract

This article provides an overview of the composition and purpose of record-keeping in dental practice and in the context of a General Dental Council (GDC) fitness to practise (FtP) investigation. It includes an overview of the GDC's FtP process. The importance of making appropriate patient records from a dento-legal perspective is also highlighted, together with changing trends in record-keeping in the United Kingdom over the past 50 years and factors that have contributed to ‘note bloat' - a term that describes unnecessarily lengthy (at times, superfluous) records. It calls for a shift in current practice and explores alternative options for record-keeping in a more concise way and discusses how this may be achieved.

## Key points

- Presents an overview of the importance of record-keeping in dental practice and how patient records and other relevant items of correspondence are reviewed during a General Dental Council fitness to practise investigation.
- Critically evaluates current trends in record-keeping in dentistry in the UK, who really benefits from this approach, the ‘litmus test for reasonable dental record-keeping,' and the evolution of the phenomenon of ‘note bloat'.
- Emphasises the need to review and change current habits with record-keeping.
- Advocates a more concise approach with conveying salient and relevant information.

## Introduction

Dental records, encompassing both objective and subjective patient information, extend beyond written clinical notes (Box 1).^1^^,^^2^ These records serve many purposes and fulfil various functions.^3^^,^^4^ According to its fitness to practise (FtP) statistical reports, the General Dental Council (GDC) - the statutory regulator for dental professionals across the United Kingdom (UK) - reported a 22% reduction in overall concerns raised between 2018-2023.^5^^,^^6^ Notably, approximately half of the concerns raised with the GDC in the past three years have originated from, or on behalf of, patients, and most relate to clinical or conduct-related issues.^6^

In contrast, complaints about dental services in England have surged by two-thirds between 2017/18 and 2022/23.^7^ According to the Parliamentary and Health Service Ombudsman, common areas of concern include access to NHS (National Health Service) dentistry, treatment and fees.^7^ Given dentists' growing fears of litigation and the subsequent impact on their practice,^8^^,^^9^ it is unsurprising that many UK-based dental professionals are adopting an overly defensive approach to their clinical care. Indeed, Hellyer and Radford suggest that dental professionals are increasingly limiting their practice to no treatment or low-risk treatments due to a perceived increase in litigation risks.^10^ Accepting the critical role that clinical records play in defending against clinical negligence claims or allegations of professional misconduct, UK dental professionals invest substantial time, effort and resources to maintain detailed patient records.^3^^,^^4^^,^^11^ However, these records may occasionally contain excessive, non-essential information that can distract from and obscure important aspects of the patient's care.

This article examines the purpose of dental record-keeping, highlights key record-keeping requirements and explores how the GDC evaluates records during clinical investigations. A clear understanding of this topic may help alleviate some of the anxiety surrounding dental regulation, also highlighted in the GDC's 2019 publication, Moving upstream.^12^ We will also discuss how dental record-keeping can be streamlined to ensure safe care documentation.

Box 1 To show items which make up a set of patient dental records

- Handwritten and computerised clinical notes, to include details such as data to identify the patient and further charting, such as hard tissue charting and periodontal charting
- Medical history forms
- Radiographs, scans and other imaging records
- Digital scans
- Photographs and videos
- Models, to include study models, working models and diagnostic wax-ups/tooth set-ups
- Correspondence with patients/carers and other healthcare professionals/providers, to include referrals correspondence
- Recording of any telephone conversations
- Written treatment plans
- Consent forms
- Statements relating to custom made devices under the Medical Devices Regulations

## The purpose of dental record-keeping and the requirements

In the UK, dental professionals are bound by legal, professional and ethical obligations regarding the creation and maintenance of dental records. These obligations are governed by:

- The GDC
- The Care Quality Commission (CQC)
- Healthcare Inspectorate Wales
- The Regulation and Quality Improvement Authority (RQIA)
- Scottish Government Records Management Code of Practice for Health and Social Care (Scotland) 2020
- Part 13 of the model NHS dental contract and the NHS General Dental Services contract
- The Data Protection Act 2018 and the General Data Protection Regulation (GDPR).

According to the GDC's Standards for the dental team (2013),^13^ registrants must make and keep contemporaneous, complete and accurate patient records. Patient records are essential for understanding oral health, tracking patient progress, guiding clinical decisions, helping to ensure continuity of care, supporting audits and research, and for the recording of the consent process. They may also be required for forensic purposes. However, it is important to stress that comprehensive clinical records must be contemporaneous, complete and accurate. This is distinguished from records that are voluminous, containing superfluous information and lacking in the key facts that will help to support patient care being provided in a safe and effective manner. 

Accurate records promote patient safety and help avert errors, such as those arising from notational inconsistencies which could lead to treating the wrong tooth; although, it is accepted that minor errors in record-keeping may not necessarily harm patients. We wish to draw particular attention to the deductive reasoning flaw which supports the adage that ‘if an event or intervention is not recorded, then it didn't happen'. This falsehood should be approached with nuance as unrecorded actions may have taken place. In the words of Sarah Przybylska, a barrister who represents healthcare professionals facing regulatory proceedings: ‘close examination of most patient notes will show that on occasion, things are done but not recorded or are recorded in some parts of the notes (on a chart, perhaps) but not elsewhere'.^14^ It might be the case that where excessive, superfluous, or template notes are used indiscriminately, their presence may not necessarily reflect what did happen.

Clinicians may be tempted to falsify records to cover up errors, inflate claims for third-party payment, or manipulate patient data to meet performance targets and comply with legal and regulatory requirements. The alteration, intentional falsification, addition, or deletion of clinical records to misrepresent events and/or interventions is indefensible and carries severe legal, dental and ethical consequences. If inaccurate information is discovered in the records at a later date, the appropriate remedy is to add an addendum noting the inaccuracy. The key elements of dental record-keeping have been summarised in Table 1.

**Table 1:** A summary of the key elements of dental record-keeping

Accuracy and clarity	All entries must be precise and unambiguous, ensuring that the information is correct and easily understood by other healthcare professionals who may be involved in the patient's care. Avoid unnecessary jargon and ensure clarity in all documentation
Comprehensive documentation	Records should cover all essential aspects of patient care, including medical and dental history, diagnosis, treatment plans, procedures and outcomes. They must also include discussions of risks, benefits, alternatives, patient preferences, and any follow-up instructions, advice or care provided. It is also prudent to record the patient's compliance to advice - from a dento-legal perspective, a non-compliant patient helps to advance the contributory negligence argument - for example in cases relating to periodontal disease progression
Timeliness	Documentation should be completed as soon as possible after the patient interaction to ensure accuracy and prevent memory gaps. Any delay in writing of the record increases the risk of omitting important information.^11^ This helps maintain a contemporaneous record (we take ‘contemporaneous' to mean writing the notes before the next patient is seen in the dentist's appointment schedule) which is vital in defending the care provided in any future legal or regulatory review especially when addressing concerns that arise long after care has been provided. While civil claims must be filed within three years of the alleged negligence or when the patient becomes aware of it, GDC investigations have no time limit
Legibility	Whether handwritten or digital, records must be easily readable. Poor handwriting or unclear digital formatting can lead to misinterpretation and compromise patient safety or legal defence
Confidentiality	Patient records must be stored securely and accessed only by authorised personnel, in compliance with data protection laws like General Data Protection Regulation. This ensures patient privacy and builds trust in the healthcare system
Continuity of care	Ensure that records support continuity by documenting all relevant communications, referrals, handovers and follow-up appointments. This ensures that other healthcare providers can seamlessly continue the care of the patient based on the documented history
Audit	In digital systems, every entry, edit and amendment should be tracked with an audit trail showing who made the change, when and why. This transparency ensures the integrity of the records and helps identify any unauthorised alterations
Professional responsibility	The treating clinician is ultimately responsible for ensuring that the records accurately reflect the care provided. Even if assistants or other staff are involved in record-keeping, the clinician must review, verify and amend the records as necessary
Consent and communication	Documenting informed consent is crucial. This not only involves recording that consent was given but also ensuring the patient's understanding of the risks, benefits and alternatives discussed. Discussions must be noted to protect against potential future complaints or legal challenges

Comprehensive dental records are essential in helping to establish the facts in dento-legal cases. They provide a detailed account of events, including the differential diagnosis, the process of obtaining informed consent and details and documentation of financial transactions.^3^^,^^4^^,^^11^ These records enable others to clearly understand what treatment was provided, as well as how, when and importantly, why it was performed.^4^ Inadequate or incomplete entries can compromise the defence argument, making it difficult, if not impossible, to defend a case, even if the actual treatment was performed to a high standard.^4^ Our collective experience in dento-legal matters suggests that it is often the ‘why?' - the rationale behind clinical decisions that is frequently omitted.

### Trends in dental record-keeping

Over recent decades, the minimalist approach to record-keeping in the 1970s and 1980s has gradually evolved into the more expansive practices we see today.^11^ Dental professionals in the UK frequently comment on the significant amount of time required to write clinical notes. Based on peer review and inter-colleague discussions, it is estimated that a clinician may typically spend between 5-10 minutes writing the records for each patient. With a daily schedule of approximately 15-20 patients, this can equate to approximately two hours per day solely dedicated to record-keeping. For a full-time dental practitioner, this amounts to nearly one full working day per week. This extensive time commitment impacts service delivery, reduces access to timely dental care and incurs financial costs.^15^

To save time, practitioners have come to rely on templates and custom screens. As a result, record-keeping has increasingly become a box-ticking exercise. This serves little purpose other than creating a superficial sense of compliance, lacking meaningful context or significance. This approach raises the question: who truly benefits from this practice? In our view, the purpose and practice of record-keeping now requires serious and urgent reconsideration. While there is a UK-wide professional obligation to maintain complete records and ideally capture as much detail as possible, we advocate a shift toward conciseness, focusing on salient and relevant information. This can be accomplished by reference in the patient records to a written, standard operating procedure (SOP) to avoid an increasingly common phenomenon referred to as ‘note bloat' - a euphemism for excessively long notes.'^16^ The latter is also a significant unintended consequence of the transition from paper to electronic health records and can arise from several factors, which include:

- Overly detailed notes - including unnecessary information that doesn't add value to patient care
- Copy-paste and template overuse - reusing prior entries or templates without tailoring them to the current visit
- Writing overly comprehensive notes to protect against potential legal challenges, often at the expense of clarity and conciseness
- Prompts - filling out every field or template, even when not clinically relevant
- Inadequate training - insufficient guidance on record-keeping
- Some clinicians may habitually include excessive detail or repetitive information in their notes, often due to influences from their training or prior experiences.

Given that registrants are likely to be most apprehensive about an investigation by their regulator, it is important to understand the importance and relevance of clinical records for GDC FtP investigations. At a minimum, this may help to clarify commonly held unhelpful misconceptions.

### Who are dental records for?

A broad range of authorities, in addition to the patient, can access clinical records. The prospect of multi-agency scrutiny of the records cultivates a climate of fear, causing dentists to be overly cautious. This often results in the misguided belief that prioritising quantity over clarity in record-keeping is an effective way to reduce the risk of criticism or accusations of incomplete documentation. As noted in a 2024 GDC blog post,^17^ the practice of documenting everything in minute detail seems to be primarily motivated by concerns over potential complaints or claims, as well as the repercussions of inadequate documentation to demonstrate what occurred during an appointment.

Comprehensive records must not be confused with voluminous records that are lacking accuracy and completeness. Such excessive entries lead to information overload, which not only hinders clinician efficiency and may impair understanding, but also increases the likelihood of overlooking critical information that could be buried within the records. This information may be vital for patient safety, such as relevant medical conditions or the confirmation of consent. It is important to get this balance right.

In reality, the vast majority of clinical record entries are read only by the treating dentist who is the author and occasionally by colleagues involved in shared care. Other clinicians need to rely on the records and be sure what is in them properly reflects the discussion, warnings and record of what was done. If the records clearly outline what needs to be done next and why, and are easily understood by your colleagues, logically, this should be the ‘litmus test' of what is reasonable to record, nothing more.

### Record-keeping guidance

The GDC sets standards for conduct, performance and ethics within the dental team. According to the GDC's Standards for the dental team,^13^ dental professionals are required to provide high-quality care based on current evidence and best practices. However, it is important to note that the GDC does not set clinical standards. Standards for record-keeping are also established by the CQC in England, the RQIA in Northern Ireland^18^ and other bodies in Scotland and Wales, as listed above.

Guidance to assist with decision-making in specific situations and how dentistry should be practised and recorded was first published in 2001 by the Faculty of General Dental Practice of the Royal College of Surgeons (UK) (FGDP [UK]), now the College of General Dentistry. These guidelines were updated in 2009 and again in 2016.^19^ It is essential to recognise that these guidelines are based on expert opinion and were never intended to serve as rigid shackles on clinical practice.^19^

Newton *et al*. (2019),^20^ employed a four-stage Delphi method to reach a consensus on a list of core items to be recorded during new patient appointments, recall appointments and emergency dental appointments in primary care. The findings from this study were subsequently adopted by NHS England (NHSE) and NHS Improvement to create unified Dental record-keeping standards.^21^ While these standards were expected to be widely accepted, their implementation has been hindered by inconsistent terminology, conflicting advice across publications and a general lack of awareness. One notable example of conflicting advice involves the requirement to perform and document an occlusal assessment during new patient examinations and extra-oral examinations during recall appointments. While the guidance deems these assessments as ‘basic recommendations,' the NHSE standards categorise them as ‘aspirational' rather than ‘essential'.

Beyond these specific standards, other laws, regulations and guidelines influence clinical examination and record-keeping. These include, but are not limited to, the Ionising Radiation (Medical Exposure) Regulations (IRMER) and recommendations from the National Institute for Health and Care Excellence regarding risk assessments for certain oral diseases, which also help determine appropriate recall intervals.^22^^,^^23^

However, adhering to guidelines without considering the clinical context can reduce clinician autonomy, merely following rules rather than exercising professional judgement that is specific to each patient's needs.^24^ Legally, ‘guidance' refers to non-binding recommendations or advice issued by regulatory or professional bodies. Although not enforceable by law, guidance is highly influential in the professional setting and can be used by courts and regulatory bodies to assess whether a professional's care has met accepted standards. Failing to follow guidance may result in disciplinary actions from regulatory authorities, even if it doesn't constitute a legal violation. Moreover, in legal cases, guidance is often referenced to determine whether a professional's actions were appropriate.

There is also a risk that guidelines and standards may be misapplied by third parties for purposes beyond their original intent. For instance, it has been suggested the GDC has sometimes used the aspirational FGDP standards as benchmarks for assessing misconduct, while claimant lawyers have applied them to the Bolam test in clinical negligence claims.^25^

### The GDC FtP process

The primary purpose of the GDC is to protect patient safety and maintain public confidence in the dental profession. When a concern is raised by an informant with the GDC about a dental registrant's fitness to practise, it will initially be screened to determine if the matter should be pursued. Details of the four stages of the FtP process and the tests applied have been included in Table 2. Cases that satisfy the initial assessment test (Stage 1) will generally progress to Stage 2 (assessment). For clinically related cases at the latter stage, a clinical advisor may be instructed to provide a written opinion (report) to determine if the standard of care provided during a stipulated time frame has met the level of professional practice reasonably expected of a registrant working within the same discipline. This will help the GDC to decide whether the case raises an allegation that the dental professional's fitness to practise is impaired.

**Table 2:** The four stages of the General Dental Council's fitness to practise process and the tests applied. Progression will take place only when the test has been satisfied

**Stage number**	**Name**	**Test applied/outcomes**
1	Initial assessment	Does the information give rise to a concern that: Harm has been caused, or may have been caused, to a member of the public, and/or Public confidence in the profession has been, or may have been undermined?
2	Assessment	Does the conduct alleged: Give rise to a concern that harm has been caused to a member of public, or Give rise to a concern that public confidence in the profession has been or may be undermined; And the circumstances are such that the allegation is serious enough that, if proved, it raises the issue that the FtP of the practitioner may be impaired.
3	Case examiner	Is there a real prospect of a practice committee finding the facts, statutory ground of impairment and current impairment all proved?
4	Practice committee	-

Clinical advisor investigations are usually based on the appraisal of patient records and other items of correspondence; however, no direct contact with the informant takes place. This makes the clinical records and the information they contain to form a crucial element of this assessment.

The advisers report will provide an overall conclusion, with five possible outcomes:

1. At the level of professional practice reasonably expected
2. Below the expected level for some aspects of the care, but overall, the standard of practice was not unsatisfactory to the extent the overall care was below a reasonable standard
3. Below the level of professional practice reasonably expected
4. Significantly below the level of professional practice reasonably expected, or
5. There was insufficient information provided to conclude.

In forming their overall opinion, the clinical advisor must draw upon their knowledge, expertise and personal experience. While GDC clinical advisers have access to general guidelines that may indicate whether the standard of conduct or practice has fallen below reasonable expectations (as listed in Box 2), these guidelines are neither prescriptive nor exhaustive. The clinical advisors' outcomes may be influenced but not rigidly dictated by appropriate authoritative guidance (where it may not always be reasonable to expect the dental professional to consistently satisfy the requirements) and the available professional standards, as well as published examples of ‘serious incidents' and ‘never-events' in primary dental care.^26^^,^^27^^,^^28^

A blog post by the GDC in June 2024^17^ communicated the approach usually applied by their clinical advisers with the reviewing of patient records during an FtP investigation, referring to the taking of a fair, balanced, pragmatic and proportionate approach. In this post, it was noted that adhering to professional standards does not require documenting every single detail; however, it is essential for the documentation to be clear and comprehensive. Please note, however, the approach described below is not aimed at setting standards in clinical examination and record-keeping.

Clinical advisers instructed by the GDC will usually consider the available documentation in four segments:

1. The pretreatment aspects
2. Obtaining consent
3. The treatment phase, and
4. The aftercare provided.

The adviser will aim to assess the standard of care provided for a given time frame and matters raised in the complaint. The level of criticism a clinician might face for not meeting reasonably expected standards will be influenced by the type of appointment, the extent of deviation from accepted standards, and the severity of any harm or potential harm that has occurred or may have arisen. Unsurprisingly, criticism will be more severe if there is a significant departure from standard practice and/or if the patient has been harmed.

Obtaining and recording a current medical history for every patient is essential. This ensures that all relevant health information is available for safe and effective care, demonstrates adherence to expected standards, and helps mitigate the risk of criticism or liability in the event of adverse outcomes.

The patient's reason for attendance and the details of their complaint should be recorded clearly and accurately to aid diagnosis and treatment planning. Documenting socio-behavioural and past dental history is important in certain cases, but if there are no clear risks of harm, criticism from the GDC's clinical adviser is likely to be descriptive rather than substantive.

For the clinical examination, the clinical adviser will review the records to ascertain whether they support the diagnosis and treatment plan. For example, records that confirm an extra-oral assessment was undertaken to help support the prescription of antimicrobial drugs for the (adjunctive) management of an acute dento-alveolar infection with evidence of pyrexia or a diffuse swelling.^29^

Conducting and documenting intra-oral soft tissue assessments, including oral cancer screening, is generally expected at each course of care. This is crucial for early detection and diagnosis, particularly for higher-risk patients and those who are infrequent dental attenders.

Intra-oral hard tissue charting and periodontal screening (depending on the nature of the appointment and the presentation) may be relevant, but it is worth noting that using a numerical clinical index to indicate disease severity without recording the actual scores is considered to have limited value. Given that published research has shown high levels of inconsistency among dental practitioners when charting tooth wear and recording occlusal assessments (including risk assessments), it would be unreasonable to be overly critical of omissions unless these records are of material importance and relevance.^30^^,^^31^

In accordance with the IRMER, duty holders are responsible for various aspects of radiographic practice, including the clinical evaluation and interpretation of images.^22^ Clear recording of the findings and diagnosis is expected.

GDC clinical advisers may reasonably expect to see documentation of a definitive or differential diagnosis, evidence of logical treatment planning, and risk assessment outcomes for dental caries, periodontal disease and oral cancer.^23^ However, where the records may be lacking in the latter areas, but it is evident from an otherwise satisfactory set of patient records they are highly likely to have been undertaken, the likely findings applied, and the care plan is supported by a written treatment plan, the resultant level of criticism may be more limited.^17^

Failure to obtain consent is an issue that occasionally arises as an allegation during a GDC professional conduct committee hearing (Stage 4 of the FtP process) if a patient or informant alleges that consent was not valid. It is essential to show that valid consent was obtained before treatment was commenced. This includes providing patients with information they are likely to want and need to know, discussing relevant alternatives and the recommended option, clarifying whether the care is provided under the NHS or privately, and documenting their understanding of what was discussed. Such detailed records will be critical in defending against these allegations. Dentists are often unsure whether they need to document every alternative along with an exhaustive list of risks and benefits for each option. It must be clear from the records that the reasonable alternatives were given, where relevant, including a recommended option.

Consent forms, without the accompanying discussion and proper documentation, may not adequately demonstrate that valid consent was obtained. Written consent must, however, be attained for treatment involving the use of conscious sedation or general anaesthetic.^13^

For any treatment provided, the clinical adviser will aim to appraise the available evidence to verify the provision of good-quality care, delivered in a safe and effective way, adopting a reasonable and logical approach, and to include any advice given, details of any drugs prescribed and the treatment execution (including post-operative instructions etc.).

High standards of professionalism is crucial, especially when things go wrong. It is important to provide effective aftercare, timely complaint resolution, openness, honesty and apologies. Providing records of this correspondence can significantly influence the investigation's outcome by showing that the patient's interests were prioritised.

The GDC casework team will use the clinical advisor's report to decide whether to close a case or progress it to Stage 3 of the FtP process (case examiner). For closed cases, the closure letter usually details key findings, aiding postgraduate development, reflective practice and identifying learning needs. For progressed cases, the findings help draft allegations.

Box 2 Examples of aspects where a General Dental Council clinical adviser may be more critical

- Evidence of actual harm or unduly increased risk of harm to a patient, including a relatively small risk of very serious harm
- The failure observed is a gross or significant departure from the standard expected
- A breach of the law/statutory requirement
- Foreseeability (reasonably foreseeable harm - deliberate/premediated conduct)
- The failure/failures are elemental and relate to basic skills
- Cumulatively, the failings amount to a gross or a significant departure from the standard expected

### Changes with GDC FtP

As described in a further blog post by the GDC in 2024,^32^ there is often a misconception that the GDC inflates concerns out of proportion to their seriousness. Negative perceptions about the GDC may also be influenced by several factors, including (but not limited to) social media, press commentary, peers and some educators and trainers.^12^ It is important to address these unhelpful misnomers, which may contribute to the taking of a more defensive approach with patient care and an unhelpful and time-consuming reliance on voluminous notes.

## Delegation

Where records are made by other members of the dental team, they should be carefully checked by the treating clinician. If there is a delay in writing a clinical record, the reason for the delay should be clearly documented. We acknowledge the widespread use of auto-notes and pre-filled templates to save time but caution that they must be carefully reviewed to ensure accuracy.^33^ Clinical advisers and dento-legal consultants often comment on errors in template records where the copy-paste culture creates anomalies. Examples include:

- Reference to discussions about the risks of maxillary sinus exposure during the extraction of a mandibular premolar
- Periodontal probing and BPE (Basic Periodontal Examination) scores on edentulous patients
- Provision of smoking cessation advice to toddlers.

Such discrepancies prompt questions about the validity of the records and ultimately, the honesty and integrity of the dental professional involved.^33^

Serious events with potential for harm must be clearly documented. For example, if a chemical burn occurs, record the sequence of events, injury assessment, management, aftercare and communication with the patient. Always exercise the professional duty of candour when accidents arise.

Timely handling of complaints and local resolution can greatly influence investigation outcomes. Avoid reluctance to engage with indemnity providers, as it can hinder future investigations. Clinical advisor investigations are limited to provided information. If the GDC requests records, seek advice from your indemnity provider and ensure all records, both paper and digital, are disclosed, ensuring that a full picture of a complaint can be seen in context.

## Future considerations

There is the need to strike a balance between the minimalistic dental record-keeping of the 1970s and 1980s and the more detailed and extensive records commonly seen today.^11^ Finding this equilibrium ensures that notes are both concise and clinically relevant, avoiding the extremes of under- or over-documentation. This requires a paradigm shift from all stakeholders, including clinicians, regulators and administrators, to promote concise, context-specific documentation that supports patient care while meeting legal, compliance and quality requirements.

Advancements in artificial intelligence (AI), along with the development of speech-to-text software, may offer a long-term solution. Appropriate AI applications in dental record-keeping could potentially significantly enhance accuracy, help to ensure consistent documentation, streamline workflows through automated data entry and allow clinicians to focus more on patient care. The implementation of AI in dental record-keeping could represent a transformative approach and further reduce note bloat by analysing patient data trends to highlight what is relevant to treatment and patient safety, and suggest relevant content for notes based on previous entries, helping to maintain orderly, clear records and reducing unnecessary information. However, it is imperative that any professional standards and legislation are satisfied. The data protection and storage implications of these AI applications needs further consideration but this is beyond the scope of this paper.

When it comes to recording procedural details, we suggest that serious consideration should be given to the creation and adoption of SOPs alongside the clinical record. By standardising processes and defining clear, repeatable steps, SOPs reduce variation, ensure consistent patient care, improve safety and efficiency. The reference in the records to the SOP would then negate the need to record extensive details of the technical execution and help to provide a comprehensive, accurate and complete account. An example may be in conservative dentistry, where an agreed SOP for a restoration involves cavity preparation, the removal of dental caries, material selection, the use of a matrix system, and verification of the occlusion and the inter-proximal contact areas. Clinical records for a given SOP may be further tailored as necessary to describe any changes which may stipulate a deviation from specific aspects with the SOP and any further challenges that may have been encountered, etc. Such SOPs could be prepared for many procedures routinely undertaken in primary dental care. If these are evidence-based, agreed and documented, then clinicians can refer to them in the clinical record. This will reduce the volume of records and save time, and in turn, may help alleviate some of the well-documented current challenges with recruitment and retention of the workforce in the UK.^34^ It should be noted that any reference to a specific SOP means that the relevant SOP would need to be disclosed as part of the clinical record.

## Conclusion

The dental profession should set record-keeping standards. Given the current challenges in the UK dental workforce, such as stress and burnout, it's crucial to address myths about the GDC's clinical record review during FtP investigations. The focus should be on creating succinct, high-quality clinical records which demonstrate the sequence, safety and effectiveness of dental care and not on unnecessary, superfluous entries. If a review of record-keeping practices is needed to improve efficiency, safety and accuracy, dentists should be supported by all stakeholders. Lawyers will then have no choice but to apply the revised guidance. It means they will have no choice but to follow the trend rather than lead it.
